# Effects of photobiomodulation on annulus fibrosus cells derived from degenerative disc disease patients exposed to microvascular endothelial cells conditioned medium

**DOI:** 10.1038/s41598-020-66689-0

**Published:** 2020-06-15

**Authors:** Min Ho Hwang, Jae Won Lee, Hyeong-Guk Son, Joohan Kim, Hyuk Choi

**Affiliations:** 1 Department of Medical Sciences, Graduate School of Medicine, Korea University, Seoul, South Korea; 2 Department of Neurosurgery, Guro Hospital, College of Medicine, Korea University, Seoul, Korea

**Keywords:** Cell biology, Mechanisms of disease, Biological techniques, Optogenetics, Biotechnology, Tissue engineering, Spine regulation and structure, Chemokines, Cytokines

## Abstract

Intervertebral disc (IVD) degeneration with chronic low back pain is associated with neo-vascularisation into the deeper IVD regions. During this process, endothelial cells (ECs), which are primarily responsible for angiogenesis, interact with the adjacent annulus fibrosus (AF) cells, which are the first line of defence against the invasion of vascular structures into deeper IVD regions. However, the accumulation of inflammatory and catabolic enzymes that results from this interaction promotes matrix degradation and an inflammatory response. Thus, regulating the production of these mediators and catabolic enzymes could ameliorate IVD degeneration. Photobiomodulation (PBM) therapy is a non-invasive stimulation known to have biologically beneficial effects on wound healing, tissue repair, and inflammation. Here, we examined the effects of PBM, administered at various wavelengths (645, 525, and 465 nm) and doses (16, 32, and 64 J/cm^2^), on EC-stimulated human AF cells. Our results show that PBM selectively inhibited the EC-mediated production of inflammatory mediators, catabolic enzymes, and neurotrophins by human AF cells in a dose- and wavelength-dependent manner. These results suggest that PBM could be a superior and advanced treatment strategy for IVD degeneration.

## Introduction

Symptomatic intervertebral disc (IVD) degeneration is strongly associated with chronic low back pain (LBP), a condition that represents a serious socio-economic burden. Although the aetiology of IVD degeneration has not been completely elucidated, increasing evidence suggests that neo-vascularisation resulting from IVD tissue inflammation or impaired matrix homeostasis could be a major cause of symptomatic disc degeneration^1^.

IVD tissue is composed of a centrally located nucleus pulposus (NP) that is surrounded by the annulus fibrosus (AF). Fibroblast-like AF cells play a crucial role in maintaining the matrix homeostasis and turnover by regulating the extracellular matrix (ECM) enzymes such as matrix metalloproteases (MMPs)^2^. Under normal conditions, except for the outer third of the annulus fibrosus, the IVD tissue is avascular and aneural. In contrast, in patients with chronic LBP, neo-vascularisation has been observed in deeper IVD tissues including the inner AF and NP regions. Furthermore, multiple lines of evidence have shown that synthesis and ingrowth of aberrant blood vessels can be the specific end-product of IVD degeneration^3–5^. Endothelial cells (ECs) are primarily responsible for angiogenesis, which is the formation of new blood vessels from pre-existing vessels^6^. During IVD degeneration, AF cells secrete inflammatory mediators, angiogenic activators, and ECM-modifying enzymes including interleukin (IL)-6, -8, vascular endothelial growth factor (VEGF), and MMPs^1^. These molecules promote matrix destruction and trigger an inflammatory response, resulting in the formation of a physical space and/or physiological response, which allows the ECs to invade deeper into the AF region. Sprouting and invasive ECs can interact with AF cells located in the inner IVD. Furthermore, during symptomatic IVD degeneration, nerve ingrowth was reported to be closely associated with neo-vascularisation, which is thought to occur during neuropathic pain. This phenomenon is probably mediated by neurotrophins, including nerve growth factor (NGF) and brain-derived neurotrophic factor (BDNF)^7,8^. Thus, an in-depth understanding of the interaction between AF cells and ECs, and the underlying molecular mechanisms, will be critical for improving current therapies that have thus far proven ineffective. Additionally, these molecules could be therapeutic targets for the treatment of IVD degeneration accompanied by chronic LBP.

Increasing evidence suggests that photobiomodulation (PBM) therapy provides a non-invasive biophysical stimulation that has beneficial effects on processes such as wound healing, tissue repair, and inflammation^9–11^. Our previous studies showed that PBM irradiation significantly reduces inflammatory mediators and ECM-modifying enzymes such as IL-6, IL-8, and MMPs in the presence of factors produced by activated THP-1 macrophages^12–15^. These studies highlight the regulatory effects of inflammatory mediators during the early stages of IVD degeneration, which is thought to occur because of interactions between IVD and immune cells. In the current study, we focus on the effects of secreted factors in the conditioned media of ECs on human AF cells as an initial understanding of the cellular communication between human AF cells and ECs. Furthermore, we tested the effects of PBM on human AF cells exposed to conditioned media of ECs as novel therapies for regulating biological molecules.

## Results

### Morphological characterisation of human microvascular endothelial cell (HMEC)-1 and effect of EC-conditioned medium (ECCM) on the degenerative response in human AF cells

**Figure 1:**
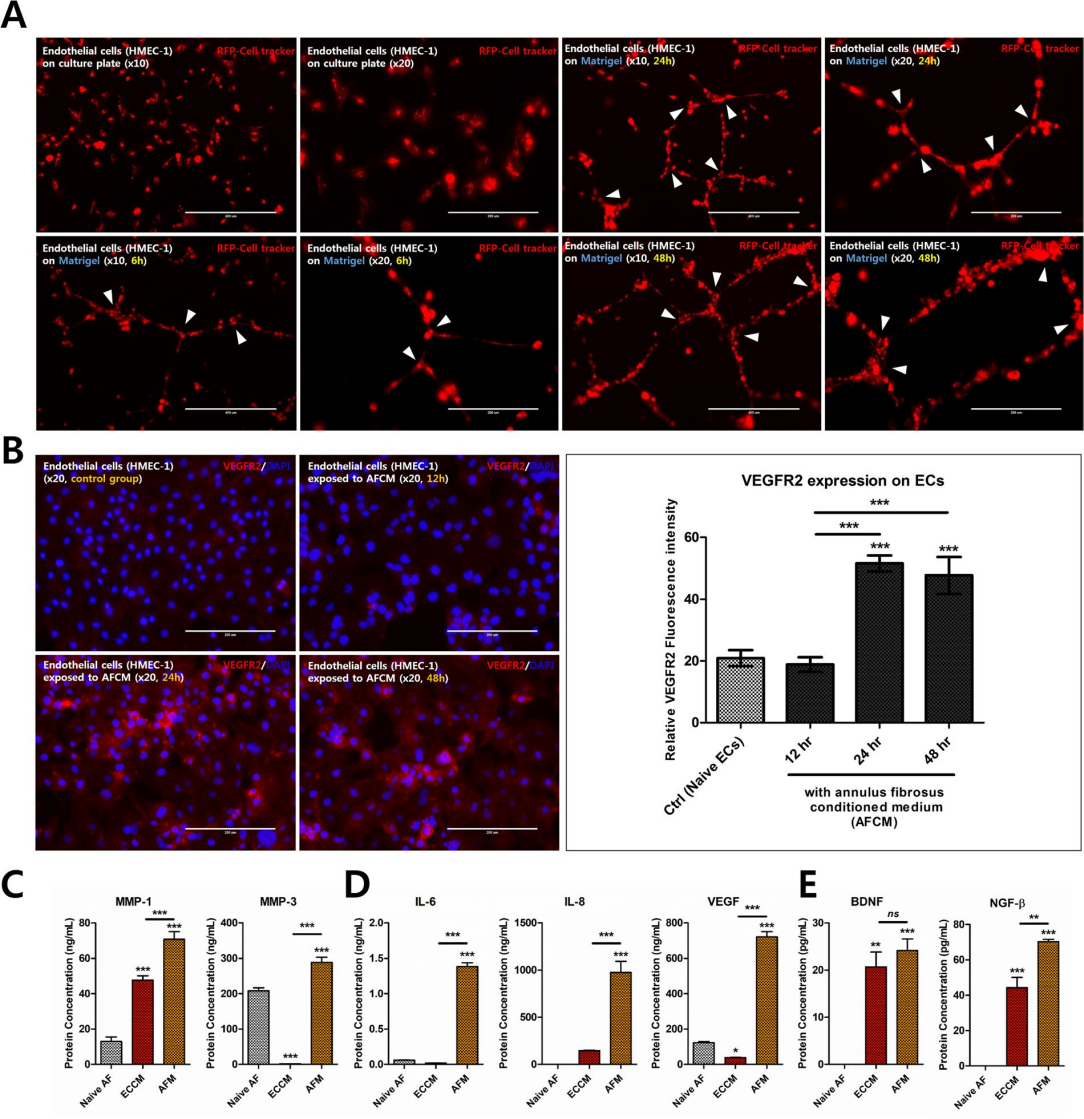
Morphological characterisation of HMEC-1 and the effect of endothelial cell-conditioned medium on catabolic enzymes, inflammatory mediators, and neurotrophins production by AF cells. (**A**) HMEC-1 was cultured in Matrigel for 48 h. Immunofluorescence images show capillary-like structures and a branching network (white arrowhead) after 6 h. (**B**) Immunofluorescence and quantitative fluorescence intensity expression of VEGFR2 in HMEC-1 exposed to AF conditioned medium (AFCM). (**C**) Production of ECM-modifying enzymes, (**D**) inflammatory mediators, and (**E**) neurotrophins by human AF cells stimulated by ECCM. *p < 0.05, **p < 0.01, ***p < 0.001, ns, no significant difference, compared to naïve AF cells. Line indicates a comparison within each group. Human AF cells were isolated from the disc tissues of seven patients and used at passage 2. Scale bar = 400 μm (×10) and 200 μm (×20). Naïve AF, human AF cells cultured in basal medium; ECCM, conditioned medium derived from endothelial cells cultured in basal medium; AFM, human AF cells cultured in ECCM; AFCM, conditioned medium derived from human AF cells cultured in basal medium.

First, we sought to verify that the HMEC-1, used in this study as a model endothelial cell line, maintain their phenotypic features and form capillary-like structures in culture. The cells were cultured in Matrigel for 48 h and began to form capillary-like structure after the first 6 h. They continued to form cohesive and looped branching networks for the 48 h culture period (Fig. 1A),indicating that the HMEC-1 exhibit all the morphological characteristics of ECs. Immunofluorescence imaging revealed high levels of cytoplasmic VEGFR2 expression in HMEC-1 that were exposed to AFCM for 24 to 48 h. Quantitatively, the VEGFR2 expression calculated from the average red fluorescence intensity in HMEC-1 was shown to have an increasing trend by AFCM at 24 and 48 h (Fig. 1B). These results show that the HMEC-1 used in the present study exhibits the key phenotypic feature of endothelial cells. Hence, the data obtained using HMEC-1 in this study are reliable.

To investigate the expression of inflammatory mediators, catabolic enzymes, and neurotrophins in human AF cells after exposure to ECCM, the productiCon of IL-6, -8, VEGF, MMP1, MMP3, NGF, and BDNF protein was measured by enzyme-linked immunosorbent assay (ELISA). Human AF cells cultured in ECCM secreted significantly higher levels of catabolic enzymes (Fig. 1C), pain-related/pro-angiogenic factors (Fig. 1D), and neurotrophins (Fig. 1E) than both naïve human AF cells and naïve ECCM. In addition, VEGFR2 was measured in HMEC-1 cultured in AF conditioned medium (AFCM). These results show that factors produced by ECs induce the release of various mediators and catabolic enzymes from human AF cells, which is thought to occur during the progression of IVD degeneration and neo-vascularisation.

### Effect of PBM on ECM-modifying enzymes in ECCM-stimulated human AF cells

**Figure 2:**
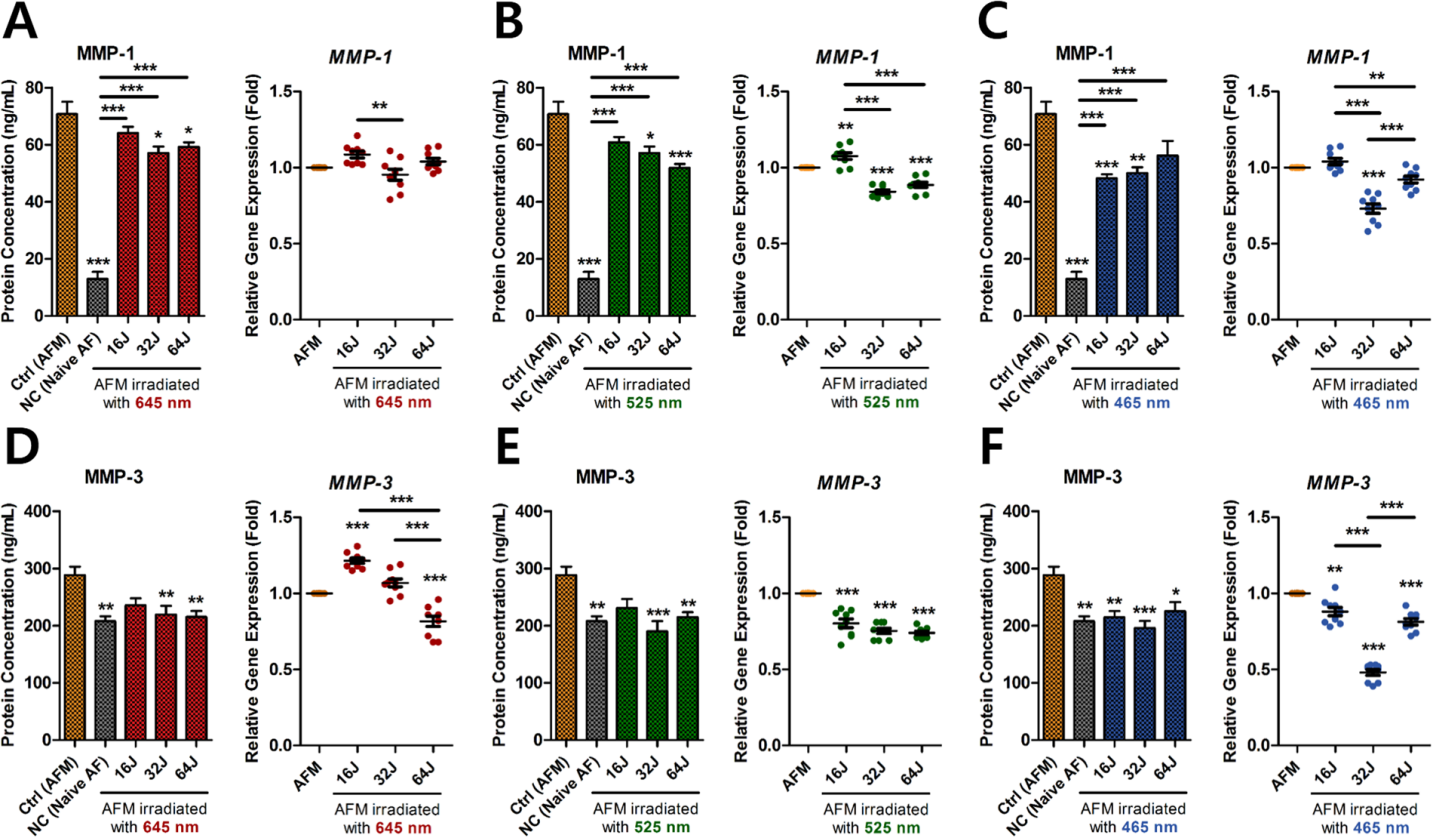
Gene and protein expression of ECM-modifying enzymes in ECCM-stimulated human AF cells. (**A**) MMP1 protein production and relative gene expression at 645 nm, (**B**) 525 nm, and (**C**) 465 nm. (**D**) MMP3 gene and protein expression at 645 nm, (**E**) 525 nm, and (**F**) 465 nm. Values are mean ± SE of four or five independent experiments. *p < 0.05, **p < 0.01, ***p < 0.001, ns, no significant difference, compared to AFM. Line indicates a comparison within each group. AFM, human AF cells cultured in ECCM; NC, negative control (Naïve AF cells).

Excessive production of catabolic enzymes including MMPs by human AF cells allows ECs to invade deeper into IVD tissues via cellular matrix degradation. Thus, we evaluated the gene and protein expression of these enzymes in ECCM-stimulated human AF cells with PBM therapy at a range of wavelengths (645, 525, and 465 nm) and doses (15, 32, and 64 J/cm^2^). For MMP1 expression, also known as collagenase-1, all wavelengths of PBM significantly suppressed protein production by human AF cells exposed to ECCM (AFM) (Fig. 2A–C). All wavelengths of PBM at 32 J/cm^2^ had inhibitory effects on MMP1 production. Similarly, PBM at 525 and 465 nm at 32 J/cm^2^ decreased *MMP1* mRNA expression but was not affected by PBM at 645 nm (Fig. 2B,C). Additionally, our results showed that PBM modulated MMP3 mRNA and protein expression at all the wavelengths tested, in a dose-dependent manner (Fig. 2D–F). At 525 and 465 nm, *MMP3* mRNA was significantly down-regulated by PBM at all the doses applied (Fig. 2E,F). Except for PBM at 645 nm with 16 J/cm^2^, all doses and wavelengths of PBM significantly suppressed MMP3 protein production relative to AFM without PBM. Although PBM at a dose of 16 J/cm^2^ and 645 nm up-regulated *MMP3* mRNA expression, protein production was not changed (Fig. 2D). Interestingly, all wavelengths of PBM at 32 J/cm^2^ decreased both MMP1 and MMP3 protein production.

### Effect of PBM on mRNA expression of pain-related and chemo-attractive cytokines in ECCM-stimulated human AF cells

Higher level of IL-6 expression was found in herniated discs from patients with chronic sciatic pain than in patients with painless scoliosis. The chemokine IL-8 (also known as CXCL8) promotes neutrophil recruitment to damaged tissue and induces angiogenesis in ECs. Thus, targeting of IL-6 and IL-8 production could have beneficial effects on nociceptive pain development and excessive catabolic response, respectively.

**Figure 3:**
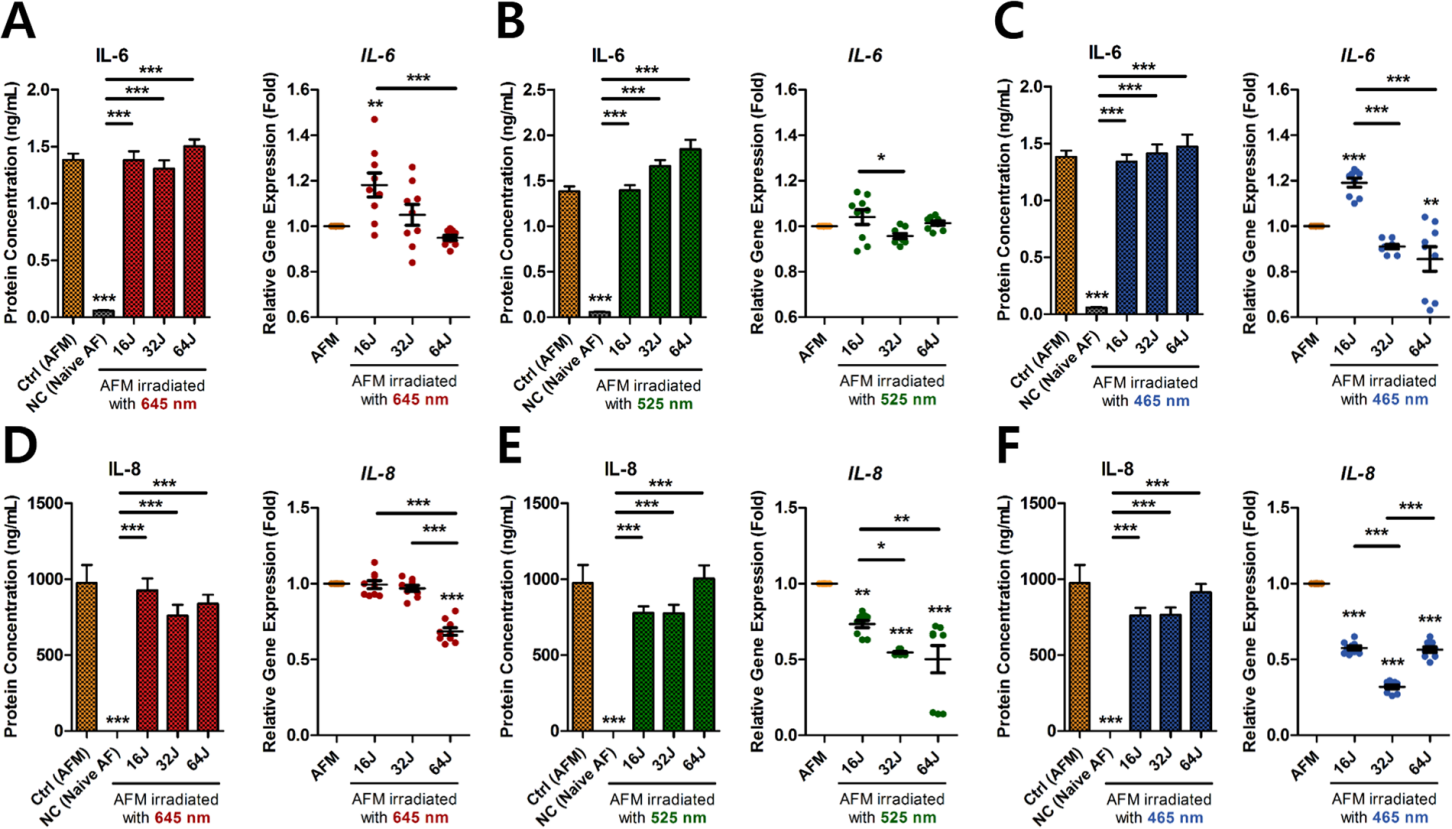
Gene and protein expression of inflammatory mediators in ECCM-stimulated human AF cells. (**A**) IL-6 protein production and relative gene expression at 645 nm, (**B**) 525 nm, and (**C**) 465 nm. (**D**) IL-8 gene and protein expression at 645 nm, (**E**) 525 nm, and (**F**) 465 nm. Values are mean ± SE of four or five independent experiments. *p < 0.05, **p < 0.01, ***p < 0.001, ns, no significant difference, compared to AFM. Line indicates a comparison within each group. AFM, human AF cells cultured in ECCM; NC, negative control (Naïve AF cells).

Production of IL-6 protein was not significantly altered in cells exposed to PBM at any of the tested wavelengths, except for 525 nm with 64 J/cm^2^ (Fig. 3A–C). PBM at 465 nm with 64 J/cm^2^ down-regulated *IL-6* mRNA expression (Fig. 3C). In contrast, compared to AFM without PBM, all the applied wavelengths significantly lowered *IL-8* mRNA expression in a dose-dependent manner (Fig. 3D–F). Although IL-8 protein production did not change significantly following PBM irradiation, the levels pointed towards a dose-dependent decrease.

### Effect of PBM on total VEGF production and VEGF subfamily mRNA expression in ECCM-stimulated human AF cells

Angiogenesis is strongly associated with nerve growth, which is responsible for pain development in IVD degeneration with chronic LBP. VEGF, a pro-angiogenic activator of ECs, promotes neo-angiogenesis during degenerative IVD progression by acting on ECs present in the outer AF and in damaged sites.

**Figure 4:**
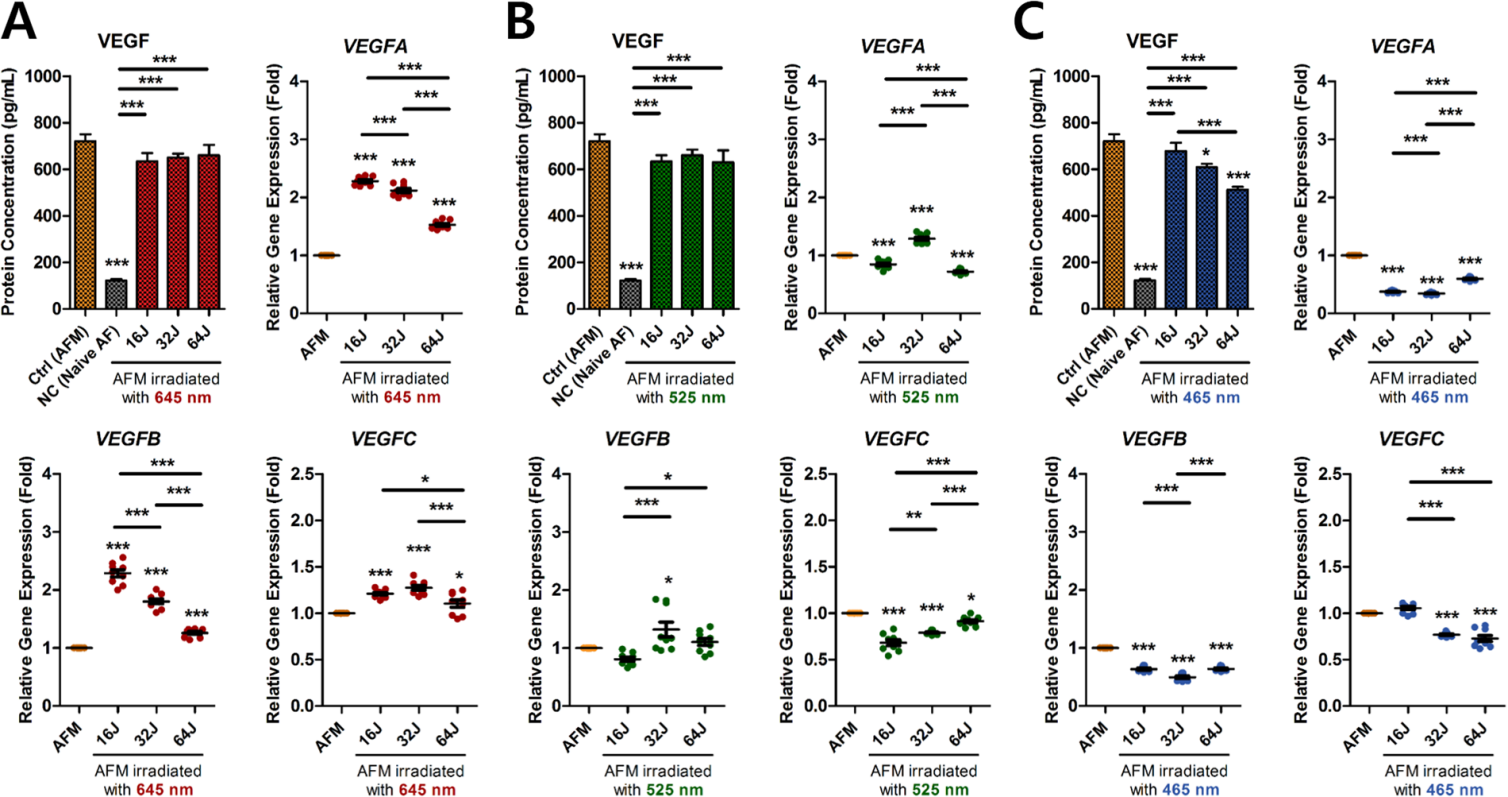
Total VEGF protein production and *VEGF* subfamily gene expression in ECCM-stimulated human AF cells with PBM. (**A**) Gene and protein expression of VEGF at 645 nm, (**B**) 525 nm, and (**C**) 465 nm. Values are mean ± SE of four or five independent experiments. *p < 0.05, **p < 0.01, ***p < 0.001, ns, no significant difference, compared to AFM. Line indicates a comparison within each group. AFM, human AF cells cultured in ECCM; NC, negative control (Naïve AF cells).

Total VEGF protein and VEGF subfamily mRNA expression were measured in the conditioned medium of human AF cells cultured in ECCM, with PBM irradiation. PBM at 465 nm with 32 and 64 J/cm^2^ significantly suppressed total VEGF protein production in AFM, which was not affected at any other wavelengths or doses (Fig. 4A–C). Similarly, PBM at 465 nm had an inhibitory effect on VEGF subfamily mRNA expression, except for at 16 J/cm^2^ on *VEGFC* expression (Fig. 4C). In contrast, PBM at 645 nm at all the tested doses significantly up-regulated VEGF family mRNA expression, although it did not significantly alter protein production (Fig. 4A). PBM at 525 nm had suppressive effects on *VEGFA* and *VEGFC* mRNA expression in a dose-dependent manner (Fig. 4B).

### Effect of PBM on pro-inflammatory cytokine and neurotrophin expression in ECCM-stimulated human AF cells

Pro-inflammatory cytokines such as tumour necrosis factor (TNF) and IL-1 are secreted by both human AF cells and immune cells. These factors promote the expression of catabolic genes, leading to an imbalance between catabolism and synthesis, which in turn results in impaired tissue integrity and pain. In a painful disc, nerve fibres accompany microvascular blood vessels as they grow into deeper IVD tissues. This phenomenon is supported by neurotrophins such as NGF and BDNF.

**Figure 5:**
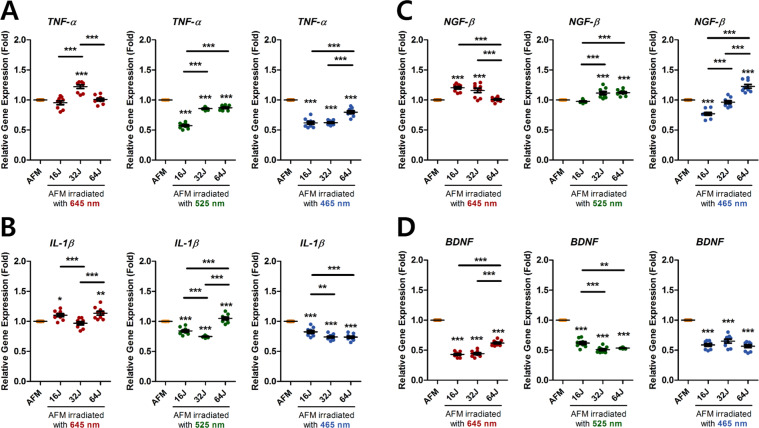
Gene expression of pro-inflammatory cytokines and neurotrophins in ECCM-stimulated human AF cells after PBM. (**A**) Relative gene expression of pro-inflammatory cytokines TNF-α and (**B**) IL-1β in AFM after PBM. (**C**) Relative gene expression of neurotrophins NGF-β and (**D**) BDNF in AFM after PBM. Values are mean ± SE of four or five independent experiments. *p < 0.05, **p < 0.01, ***p < 0.001 compared to AFM. Line indicates a comparison within each group. AFM, human AF cells cultured in ECCM.

Our results showed that PBM at 525 and 465 nm at all the tested doses effectively inhibited *TNF-α* and *IL-1β* mRNA expression in ECCM-stimulated human AF cells, except for 525 nm with 64 J/cm^2^ (Fig. 5A,B). Exposure of ECCM-mediated human AF cells to PBM at 465 nm with 16 J/cm^2^ down-regulated *NGF-β* mRNA expression, although other wavelengths and doses did not positively affect *NGF-β* mRNA expression (Fig. 5C). Conversely, all the tested wavelengths and doses dramatically suppressed *BDNF* mRNA expression in ECCM-stimulated human AF cells (Fig. 5D).

### Cytotoxicity testing using lactate dehydrogenase (LDH) secretion in ECCM-stimulated human AF cells after PBM therapy

**Figure 6:**
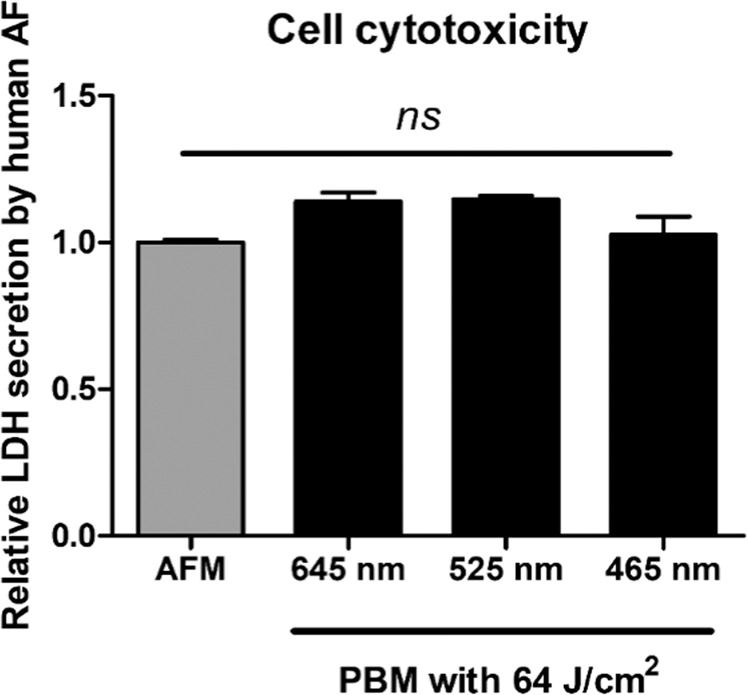
Lactate dehydrogenase release (LDH) assay used to assess LDH released by ECCM-stimulated human AF cells. The assay was performed after PBM at 64 J/cm^2^. None of the wavelengths tested were cytotoxic to human AF cells. ns, no significant difference. AFM, human AF cells cultured in ECCM.

**Figure 7:**
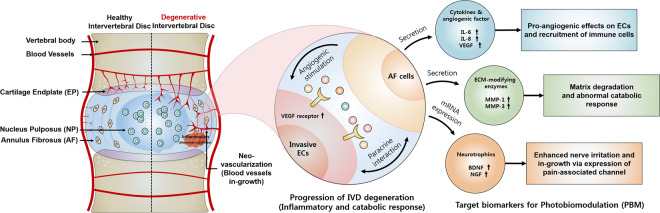
Schematic summary of the progressive IVD degeneration *in vitro* model proposed in this study. Human AF cells stimulated by factors produced by ECs secrete various factors including inflammatory mediators, ECM-modifying enzymes, and neurotrophins. ECs stimulated by AF cells express the VEGF receptor. These factors represent potential therapeutic targets of PBM therapy for the treatment of IVD degeneration.

To ensure that the results observed following PBM irradiation were not due to cytotoxicity, we performed an LDH release assay. We measured LDH secretion by ECCM-stimulated human AF cells with PBM at 64 J/cm^2^, which was the maximum dosage used in this study. We found that LDH release from ECCM-stimulated human AF cells was not significantly increased after PBM at any of the wavelengths tested (Fig. 6).Figure 7 shows a schematic diagram of the progressive IVD degeneration *in vitro* model based on the current study and the effects of PBM on human AF cells (Fig. 7 and Table 4).

## Discussion

We previously reported that PBM inhibits macrophage-mediated up-regulation of inflammatory mediators and ECM-modifying enzymes in human intervertebral disc cells in the early stages of IVD degeneration^12–15^. In the present study, we investigated the effects of PBM therapy on human AF cells exposed to factors produced by microvascular endothelial cells, which is thought to occur during the progression of degenerative IVD.

Although it is known that angiogenesis is closely linked to symptomatic IVD degeneration, few studies have investigated the interactions between human IVD cells, and ECs which are primarily responsible for angiogenesis. Moreover, the potential anti-angiogenic and anti-inflammatory effects of PBM therapy have not been studied in detail. We focused on biomolecules produced by human AF cells stimulated with potential contributing factors derived from ECs and show that PBM ameliorates the hostile microenvironment of EC-mediated degenerative IVD by modulating catabolic and pro-angiogenic molecules, at both the mRNA and protein level.

Painful IVD degeneration is characterised by the angiogenesis of microvascular structures, which accompany nerve fibres into deeper IVD tissues^1,3,16–18^. After mechanical trauma, infection, or because of genetic disease, IVD tissues promote the recruitment of circulating immune cells into damaged areas to drive endogenous repair using vascular structures located in the outer third of the AF region. However, excessive interaction between immune cells and IVD cells results in the uncontrolled production of catabolic and inflammatory factors^19,20^. This can result in spinal instability and structural defects such as matrix degradation due to an up-regulation of MMPs^21,22^. It can also induce an angiogenic response in ECs via the production of IL-8 and VEGF which are pro-angiogenic factors secreted by AF cells^23^. This cascade of events then creates a permissive microenvironment for angiogenesis and induces a degenerative condition in IVD.

Our results indicate that ECCM-induced human AF cells produce and secrete high levels of various pro-angiogenic and catabolic factors including IL-6, IL-8, VEGF, MMP1, and MMP3, suggesting that the degenerative condition in human AF cells is induced by factors produced by ECs. Our immunofluorescence data showed that ECs exposed to AFCM express VEGFR2, which is thought to promote endothelial cell function by binding soluble VEGF. VEGF is a major pro-angiogenic factor and responsible for activating ECs into tip cells^24^. IL-8, a member of the CXC-chemokine ligand (CXCL) family, is also thought to induce angiogenesis by binding the CXC receptor 2 (CXCR2) expressed on ECs and is also associated with immune cell chemotactic activity during the inflammatory response^25^. To date, three major VEGF receptors have been identified, VEGFR1 (also known as Flt-1), VEGFR2 (also known as KDR or Flk-1), and VEGFR3 (also known as Flt-4). Of these, VEGFA/VEGFR2 is the most prominent ligand-receptor complex and drives proliferation, migration, survival, and new vessel formation in ECs. VEGF binding to VEGFR results in the autophosphorylation of specific tyrosine residues in the cytoplasmic domain of VEGFR2. Phosphorylated VEGFR2 initiates downstream signalling relevant to angiogenesis and induces cellular responses in ECs including strong mitogenic and survival signals. In contrast, such a response is not induced by VEGFA binding to VEGFR1^26^.

Besides matrix degradation, a marked increase in the expression of MMPs and a disintegrin and metalloprotease with thrombospondin motifs (ADAMTS) has been reported in IVD tissue during degeneration. Specifically, a direct correlation has been shown among active MMP1 levels, collagen matrix collapse, and capillary regression due to proteolytic digestion of interstitial collagen^1,21,27^. Consistent with this, our results showed that ECCM promotes up-regulation of both MMP1 and MMP3 in human AF cells. Active MMP3 can degrade core IVD proteins and connective matrix components in the cartilage such as proteoglycans, fibronectin, and collagen type 2, which are the major components of the NP located in the inner IVD tissue. Increasing evidence suggests that MMP3 is up-regulated in cytokine-activated inflammatory human and rat NP cells^28^. This, together with our results, suggests that MMP3 up-regulation in ECCM-mediated human AF cells indirectly affects EC invasion and migration into the inner IVD region via degradation of the cartilaginous matrix at the AF-NP interface or deeper in the NP region.

During IVD degeneration, neo-vascularisation is also associated with innervation. A recent study showed that nerve fibres accompany microvascular blood vessels into symptomatic discs, and nociceptive nerve growth into painful discs is linked to NGF production^4^. Additionally, other groups have demonstrated that β-NGF and BDNF contribute to the expression of neuronal pain-associated cation channels, such as acid-sensing ion channel 3 and the transient receptor potential cation channel in the dorsal root ganglion^29–31^. Here, we showed that human AF cells exposed to ECCM secrete β-NGF, but not BDNF. Other study has suggested that BDNF expression depends on the IVD tissue region (outer AF, inner AF, or NP), as well as pro-inflammatory cytokines such as IL-1β and TNF-α. Specifically, BDNF expression is higher in the inner AF and NP after painful vibrations than in the outer AF^32^. Thus, BDNF up-regulation may have a more important role in the human NP than in the AF. Our results showed that PBM with 525 or 465 nm at all the tested doses inhibits *IL-1β* and *TNF-α* mRNA expression in ECCM-stimulated human AF cells. Additionally, all doses and wavelengths significantly suppressed *BDNF* mRNA expression.

PBM therapy has been shown to trigger various biological and physiological events during wound healing, as well as anti-inflammatory responses and enhanced cell viability and proliferation in various diseases^9,11–15,33–36^. The p38 mitogen-activated protein kinase (MAPK) pathway is known to control MMP1 and MMP3 expression in degenerative IVD. Choi *et al*. showed that LED irradiation can inhibit the action of pro-inflammatory cytokines and activate the MAPK signalling pathway^37^. Yadav *et al*. found that PBM altered the expression of 49 ECM-related genes *in vitro*^38^. A recent study also showed that PBM reduced MMP1 and MMP13 expression in injured calcaneal tendons^39^. Similarly, our results, as well as those from previous studies^12,14^, indicate that PBM ameliorates the catabolic response in degenerative IVD by down-regulating both MMP1 and MMP3. Thus, we propose that the inhibitory effects of PBM on MMPs could be mediated by this mechanism; however, further studies are needed to confirm this phenomenon.

With regard to the regulation of inflammatory mediators such as IL-6 and IL-8, these cytokines are primarily induced by pro-inflammatory cytokines such as IL-1α or TNF-β, which are secreted within minutes in response to various stimuli^40^. However, our results showed that human AF cells exposed to ECCM, even in the absence of pro-inflammatory cytokines, can produce high amounts of IL-6 and IL-8. These findings suggest that factors produced by endothelial cells may have a pro-inflammatory effect on human AF cells. Furthermore, stimulated human AF cells could accelerate the progression of IVD degeneration through the recruitment of leukocytes or/and ECs to the damaged area. These cytokines are known to play a vital role in various diseases by providing essential protective immunity^41^. However, severe diseases are also caused by the inappropriate overexpression of these cytokines. Thus, these molecules could be a therapeutic target for treating painful IVD degeneration. Our results showed that PBM at all the tested wavelengths has an inhibitory effect on IL-8 gene expression in ECCM-stimulated human AF cells but did not affect IL-6. Others have demonstrated that PBM at 635 or 660 nm significantly inhibits IL-6 and IL-8 expression in human adipose-derived stem cells and LPS-induced gingival fibroblasts, respectively^42^. Similarly, a previous study from our group also speculated that PBM modulates both IL-6 and IL-8 during macrophage-human AF cell interaction^13^.

Together our results show that PBM selectively inhibits the ECCM-mediated production of inflammatory mediators, catabolic enzymes, and neurotrophins in human AF cells in a dose- and wavelength-dependent manner. Prior to the clinical application of PBM, more information about additional photo-acceptors and effective dosage will be required. For example, combining PBM with a light guidance system or photosensitiser to deliver sufficient energy to target tissues may be an effective strategy for clinical practice.

In conclusion, we show that factors produced by ECs induce the release of degenerative IVD-related proteins from human AF cells. Furthermore, PBM regulates these factors allowing treatment of symptomatic IVD degeneration.

## Methods

### Isolation and culture of human AF cells

Human AF cells were isolated from the disc tissues of seven patients (mean age ± SE = 48.0 ± 10.6; female:male = 3:4; Pfirrmann grade = 2‒3) during surgical procedures for degenerative spinal disease according to hospital regulations. All experimental protocols were approved by the institutional review board of Korea University Guro Hospital (KUGH17208-001). Written informed consent was obtained from all patients. All methods were carried out in accordance with relevant guidelines and regulations. The specimens were placed in Ham’s F-12 medium (Gibco-BRL) supplemented with 5% foetal bovine serum (FBS; Gibco-BRL) and 1% penicillin/streptomycin (P/S; Gibco-BRL). After washing, AF regions were dissected and digested for 60 min in F-12 medium containing 0.2% Pronase (Calbiochem, La Jolla, CA, USA), followed by incubation in F-12 medium containing 0.025% collagenase for 24 h. Isolated human AF cells were cultured in 75 cm^2^ culture flasks (VWR Scientific Products, Bridgeport, NJ, USA) in a humidified atmosphere with 5% CO_2_ at 37 °C. Human AF cells were used at passage 2.

### HMEC-1 culture and production of conditioned medium

HMEC-1 was cultured in MCDB 131 medium containing 10% FBS, 1% P/S, 10 ng/mL epidermal growth factor (Gibco-BRL), 1 μg/mL hydrocortisone (Sigma-Aldrich), and 2 mM l-glutamate in 75 cm^2^ culture flasks. Passage 8–10 cells were plated (1.0 × 10^6^ cells/flask) and cultured in Dulbecco’s modified Eagle medium:nutrient mixture F-12 (DMEM/F12) containing 1% FBS and 1% P/S for 48 h. The supernatant was collected and stored at −80 °C for ELISA and other experiments. The media collected from the HMEC-1 are defined in this study as ECCM. The HMEC-1 line was established from human dermal microvascular ECs and immortalised by transfection with a pBR322-based plasmid containing the coding region for the simian virus 40 large T-antigen.

### Culturing of human AF cells in ECCM and PBM treatment

Human AF cells were plated onto 6-well culture plates containing DMEM/F12 supplemented with 1% FBS and 1% P/S at a density of 5 × 10^4^ cells per well. After 48 h, the medium was removed and replaced with ECCM for an additional 48 h. A range of wavelengths (465, 525, and 645 nm) and doses (16, 32, and 64 J/cm^2^) were used to apply PBM to each group. After the irradiation, the medium was removed and replaced with normal culture medium for an additional 48 h. The supernatant was harvested and analysed by ELISA. We decided to follow the parameters as the previous PBM irradiation studies that have been used in wound healing, tissue repair, pain relief, and inflammation reduction. We additionally have some studies that observed that the parameters of PBM could modulate the production of IVD degeneration-related protein in human annulus fibrosus cells and nucleus pulposus, which are the cell types in IVD^12–15^. The mRNA expression was analysed by quantitative reverse-transcription polymerase chain reaction (qRT-PCR). The irradiation parameter was based on our previous studies. All irradiation experiments were performed on a clean surface at 37 °C in a humidified atmosphere with 5% CO_2_. An indium gallium aluminium phosphide (InGaAIP) light-emitting diode (LED) (645, 525, and 465 nm) (Photron Co., Ltd., Anseong-si, Gyeonggi-do, Korea) was used as a light source. PBM treatment parameters are listed in Tables 1–3.

**Table 1:** Photobiomodulation parameters.

Parameter	Value
Wavelength [nm]	645 ± 15, 525 ± 5, 465 ± 5
Operating mode	Continuous wave
Luminous flux [lm] ±10%	50, 45, 25
Average radiant power [mW]	25.01, 10.05, 12.73
Aperture diameter	0.6
Beam divergence [deg]	15
Beam profile	Top Hat shape
Lens shape	Lambertian pattern

**Table 2:** Treatment parameters.

Parameter	Value
Beam spot size at target [cm^2^]	2.78
Irradiance at target [mW/cm^2^]	Continuous wave
**Exposure duration**	
16 J [sec]	640, 1591, 1257
32 J [sec]	1278, 3183, 2515
64 J [sec]	2559, 6366, 5029
**Distance of LED probe**	
Form cell culture plate [cm]	1.8
Area irradiated [cm^2^]	9 (6-well culture plate)
Radiant exposure [J/cm^2^]	
(16, 32, 64 J)	5.76, 11.51, 23.02

**Table 3:** Experimental groups.

Experimental group	Description
(1) Control	Naïve human AF cells
(2) Endothelial cell conditioned medium (ECCM)	Potential contributing factors derived from HMEC-1 cells
(3) Degenerative conditions	Human AF cells exposed to ECCM (AFM)
(4) Degenerative conditions + phototherapy	AFM with PBM

**Table 4:** Inhibitory effects of PBM therapy on ECCM-stimulated human AF cells in this study.

Molecule	Level	645 nm			525 nm			465 nm		
		16 J	32 J	64 J	16 J	32 J	64 J	16 J	32 J	64 J
MMP-1	Protein		+	+		+	+++	+++	++	
	mRNA					+++	+++		+++	
MMP-3	Protein		++	++		+++	++	++	+++	+
	mRNA			+++	+++	+++	+++	++	+++	+++
IL-6	Protein									
	mRNA									++
IL-8	Protein									
	mRNA			+++	++	+++	+++	+++	+++	+++
VEGF	Protein								+	+++
VEGFA	mRNA				+++		+++	+++	+++	+++
VEGFB								+++	+++	+++
VEGFC					+++	+++	+		+++	+++
TNF-α	mRNA				+++	+++	+++	+++	+++	+++
IL-1β					+++	+++		+++	+++	+++
NGF-β								+++		
BDNF		+++	+++	+++	+++	+++	+++	+++	+++	+++

^+^p < 0.05, ^++^p < 0.0^1^ and ^+++^p < 0.001 compared with ECCM-stimulated human AF cells.

### Enzyme-linked immunosorbent assay (ELISA)

Concentrations of MMP1, MMP3, IL-6, IL-8, VEGF, NGF, and BDNF in the supernatants were measured using commercially available ELISA kits (R&D Systems) according to the manufacturer’s instructions.

### qRT-PCR

Human AF cells were lysed in TRIzol reagent (Invitrogen), RNA extracted, and cDNA synthesised (Life Technologies) according to the manufacturer’s instructions. qRT-PCR was performed to determine mRNA expression of *TNF-α, IL-1β, MMP1, MMP3, IL-6, IL-8, VEGFA, VEGFB, VEGFC, NGF*, and *BDNF* using a SYBR Green PCR Master mix (Applied Biosystems). The mRNA expression was quantified using the 2^−∆∆Ct^ method.

### Immunofluorescence

To determine VEGF receptor expression in ECs, cells were plated onto glass-bottomed confocal plates and exposed to AF conditioned medium (AFCM) for 48 h. Human AF cells were isolated and cultured in the nutrient mixture F-12 supplemented with 10% FBS and 1% P/S. After three days, the culture media was altered to MCDB131 medium containing 1% P/S, 2 mM L-glutamate, and 1% FBS. This was cultured for an additional 48 h. The medium collected from AF cells has been defined in this study as AFCM. ECs were then fixed with 3.7% paraformaldehyde and permeabilised with 0.2% Triton X-100 in phosphate buffer saline (PBS) for 30 min and 15 min at room temperature, respectively. Cells were blocked with 5% bovine serum albumin (Millipore) in PBS, incubated with a KDR/Flk-1 VEGFR2 primary antibody (1:100; Sigma-Aldrich) overnight at 4 °C in 5% BSA, followed by incubation with an Alexa Fluor 555-conjugated secondary antibody (1:200; Invitrogen) in PBS for 1 h at room temperature. Images were acquired using an EVOS FL auto cell imaging system (Thermo Fisher Scientific Inc., USA).

### Cell cytotoxicity and LDH release

Measurements of LDH release were performed according to the manufacturer’s instructions. After the cells were exposed to PBM (64 J/cm^2^), the media were collected to quantitate the lactate dehydrogenase release. Cytotoxicity was calculated based on controls (human AF cells treated with ECCM). If the AF cells were damaged by PBM therapy, they would show increased LDH production.

### Statistical analysis

Biological samples from seven different patients for four individual experiments with at least three replicates were used. Data were expressed as means ± 95% confidence interval (CI) for four individual experiments using independent cell cultures. One-way analysis of variance and Bonferroni’s correction post hoc test was used to assess differences among experimental groups. Normal distribution within each subgroup was assessed using a Shapiro–Wilk test. For data not exhibiting a normal distribution, the Kruskal–Wallis test with Dunn’s multiple comparison was used. All statistical analyses were performed using the SPSS software (version 21.3, IBM SPSS Statistics Inc., Chicago, IL, USA). A p*-*value <0.05 was considered statistically significant. All statistical analyses were carried out in accordance with previous studies^12–15^.

## Data Availability

The datasets generated and/or analysed during the current study are available from the corresponding author on reasonable request.
